# Perceptions of educators regarding the implementation of the health promotion programme manuals for children in schools in Makapanstad, South Africa

**DOI:** 10.4102/curationis.v38i2.1529

**Published:** 2015-11-19

**Authors:** Doriccah Peu, Sanah Mataboge, Rendani Ladzani, Lene Wessels, Karien Mostert-Wentzel, Neo Seane

**Affiliations:** 1 Department of Nursing Science, School of Health Care Sciences, University of Pretoria, South Africa; 2 Department of Human Nutrition, School of Health Care Sciences, University of Pretoria, South Africa; 3 Department of Occupational Therapy, School of Health Care Sciences, University of Pretoria, South Africa; 4 Department of Physiotherapy, School of Health Care Sciences, University of Pretoria, South Africa; 5 Department of Radiography, School of Health Care Sciences, University of Pretoria, South Africa

## Abstract

**Background:**

Health promoting schools focus on, amongst other things, preventing leading causes of death such as Sexually Transmitted Infections (STI's), Human Immune Deficiency Virus (HIV) and Acquired Immunodeficiency Syndrome (AIDS), a sedentary lifestyle and creating conditions that are conducive to health through health education.

**Aim:**

This study explored the perceptions of educators regarding implementation of the health promotion programme manuals in selected schools of the Makapanstad community.

**Method:**

A qualitative, exploratory, descriptive and contextual design was utilised in this study. Four schools were selected to participate in the study. Purposive sampling was used to select educators from these schools who were actively involved in the health promotion programme. Data collection was taken through focus group interviews. One focus group comprised of eight participants who were interviewed three times. The focus group interviews were conducted until data were saturated. Data were analysed using an adaptation of Tesch's method. The eight steps of Tesch's method enabled researchers to systematically analyse and organise data using colour coding to develop data into categories, sub-categories and themes.

**Results and conclusion:**

The themes that emerged during data analysis were: the perceptions of educators regarding health promotion programme manuals before implementation of manuals, and the perceptions of educators regarding health promotion programme manuals after the implementation of manuals. Introducing health promotion materials to the schools served as a point of departure for developing personal skills and creating a supportive environment for health in schools. The health promotion manual assisted the educators to attain appropriate health promotion information.

## Introduction

In order to comply with the United Nations Convention on the Rights of the Child, the South African Government pledged to ‘put children first’ as outlined in the Integrated School Health Policy (National Department of Health and National Department of Basic Education 2012). The policy aims to ensure that all children's rights are upheld. The Integrated School Health Policy was an inter-ministerial collaboration to address school children's health needs. Globally, orphaned school children are a vulnerable group as a result of parental absence caused by, amongst other things, HIV, AIDS and migrant work (DoH & DBE 2012). For this reason an intervention to ensure children's health promotion was provided to ensure that orphaned children would have similar rights to other children. The school health programme is a combination of services to optimise the physical, mental and social aspects of children's health (World Health Organisation [WHO] 2010). In addition, the WHO designed a global school health initiative in 1995 to ensure that the health of children and learners, school personnel, families and other members of the community are promoted through programmes provided by the schools (DoH & DBE 2012; Mulaudzi & Peu 2014; Peu *et al.* 2010). Therefore, school health promotion programmes should be based on collaborative interventions.

South Africa launched the ‘National School Health Policy’ (SHP) in July 2003 to address health delivery at historically poor schools by introducing the WHO's ‘Health Promoting Schools’ concept (Rajaraman *et al.* 2012; Shasha *et al.* 2012; WHO 1986). Internationally, the majority of school-based programmes that influence risk factors for non-communicable disease, like physical activity, yielded positive health outcomes if school and community members are involved (Saraf *et al.* 2012). Several health problems, such as teenage pregnancy, learning and reproductive problems exist amongst children who need collaborative interventions in order to overcome these problems. Moloi and Chetty (2011) reported that even though the life orientation (LO) subject was implemented, two thirds of grade 6 children in South Africa did not possess knowledge on HIV and AIDS. In addition, teenage pregnancy is on the increase amongst school children even though nationally and globally it is reported to have stabilised (Lavin & Cox 2012). Hence, South African children experience health problems despite their right to health information and services as entrenched in the amended *Children's Act* of 2005 (Republic of South Africa 2005). This *Act* states that children should be provided with structures, services and means for promoting and monitoring holistic health.

The School of Health Care Science of the University of Pretoria developed an outreach project in partnership with the rural community of Makapanstad in order to strengthen community participation, provide collaborative health promotion interventions and to prevent common health problems amongst children in the rural community of Makapanstad (Peu *et al.* 2010). Educators were trained using the health promotion manual that was developed by the university staff. The community is situated about 80 km from the City of Tshwane. Many parents are employed in the city and have to use public transport, leaving home at around 04:00 and returning at 18:00. As a result, there is little time for parental guidance on life skills which has become an important role of educators.

Therefore, it is essential that educators are equipped to provide health education for children's health promotion. For this purpose, they needed to possess an updated knowledge of common health problems amongst children. Through partnership initiatives, a Community-based Nutrition Training and Health Promotion Project was launched in 2004, and four schools were selected to participate in the project (Peu *et al.* 2010:34). Schools that participated included a high school, a middle school or secondary school, a primary school and a school for mentally challenged children. Activities covered by the project entailed: nutrition education, vegetable gardening, environmental and physical activities, remedial education for children with special needs, and sex education (Peu *et al.* 2010:34). The Health Promoting Schools Project was a follow up to the Community-based Nutrition Training and Health Promotion Project.

### Problem statement

The South African Department of Education (DoE) developed an LO subject in 2003, for Grades 4 to 12 learners, as an intervention to equip children with the necessary knowledge and ability to make informed decisions about sexuality and lifestyle (DoE 2003). However, the LO subject curriculum does not appear to provide sufficient knowledge to educators to assist them in the facilitation of health promotion to prevent common health problems amongst children, as there is no health promotion manual to guide educators. A previous study on the health educational needs of educators amongst the schools in Makapanstad conducted by the University of Pretoria, School of Health Sciences, identified the need to support educators, regarding their basic knowledge of health promotion in order to address the common health needs of children (Peu *et al.* 2010). Thus, a health promotion manual was deemed necessary for educator training.

### Aim of the study

The aim of this study was to explore and describe the perceptions of educators regarding the health promotion manuals before and after utilising them.

## Research design

A qualitative, exploratory, descriptive and contextual design was used to explore and describe perceptions of educators before and after utilising the health programme's promotion manuals. Ethical approval to conduct the study was obtained from the University of Pretoria's Research Ethics Committee (70/2011). Written informed consent was given by participants after the aim and objective of the project was explained. Participation in the study was voluntary.

### Population and sample

The study was conducted in four schools in the town of Makapanstad, situated in the North West Province. Makapanstad is a rural area situated about 80 km away from the City of Tshwane. The population selected for the study were all educators employed by the four schools who were included in the health promotion project at Makapanstad. Purposive sampling was used to select educators who were teaching the LO subject, and who used the health promotion manual. Two educators from each school, who were actively involved in rendering the health promotion programme as part of the LO subject participated in the study. Written informed consent was provided by the participants after the aim and objective of the project was explained. Participation in the study was voluntary.

In response to needs identified in the previous study, the researchers developed a health promotion programme manual for the educators and children, that were given to the four participating schools, to guide them in addressing health challenges in their schools. The manual themes were based on the prevalence of the following health problems in the area:

- family planning
- prevention of teenage pregnancy
- prevention of HIV and AIDS
- nutrition and physical activity.

The health promotion manuals were implemented over a period of two years after the researchers trained the educators to use the manuals. This study, therefore, explored and described the perceptions of the educators before and after implementation of the health promotion manuals in the selected schools in the Makapanstad community.

### Data collection

Three focus group interviews were conducted with the same group of eight participants on three different days until data were saturated. During focus group discussions, the perceptions of the educators regarding the period before the implementation of health promotion manuals as well as their perceptions after the implementation of the manuals were explored and described. The strategies of trustworthiness such as credibility, confirmability, dependability and transferability were upheld (Lincoln & Guba 1985). Member checking was utilised in order to verify the collected data. The researchers spent a month with the participants observing the use of the health promotion programme manuals during LO subject teaching in order to achieve prolonged engagement. Raw data were used to justify confirmability.

### Data analysis

The data were analysed using Tesch's method of data analysis. The eight steps of Tesch's method enabled the researcher to systematically analyse the data using colour coding to categorise transcript data by category, sub-category and themes (Creswell 2003:192). Two themes emerged during data analysis.

## Results

The two themes that emerged were, firstly, the perceptions of educators regarding health promotion programme manuals before implementing the manuals; and, secondly, the perceptions of educators regarding health promotion programme manuals after implementation thereof. Before and after implementation of the health promotion programme manual, five sub-themes were identified. These were:

1. lack of health promotion programme manuals
2. the roles of educators in health promotion
3. meeting the nutritional needs through learner participation
4. promoting health in schools by empowering educators to teach sex education
5. added roles and responsibilities of teachers.

The results from before and after the intervention were presented concurrently in order to allow the researchers to compare the similarities and differences.

### Lack of health promotion manuals

The lack of health promotion programme manuals was identified as the first sub-theme. Before the health promotion programme manuals were developed, the educators worked without any teaching and learning resources to address some of the adolescents’ reproductive health issues. The participants mentioned that they had to rely primarily on common sense and struggled to prepare lesson plans as the health promotion themes were not part of the LO subject. After the health promotion manual was introduced to the participants, they were able to use it as a reference when preparing lessons. The manual was regarded as a valuable resource for both the educators and the children. Before the use of the manual the participants reported the following, to affirm this sub-theme:

‘I would scratch up my head, cracking my mind on what to prepare … With regard to physical activity, it was just a syllabus to us.’ (Participant 3)

‘… before I received this manual, I was only working practically not referring to something … I used to struggle when I prepared my lessons for the health talks … I did not have enough information concerning reproductive health issues. I was just using my common sense, no matter how old I am.’ (Participant 7)

After the use of the manuals the participants responded as follows:

‘[*T*]his book has enlightened us with the information on adolescents and we discussed it among ourselves as female teachers … The manual has made us aware of the things that we did not know especially until we came across the University of Pretoria.’ (Participant 5)

Hence, there was a positive acceptance of the health promotion programme manuals by the participants, which they found very useful.

### The roles of educators in health promotion

Before the health promotion manuals were developed, the educators had limited knowledge about the effects of poverty on children and about how to deal with children coming from poor families. After utilising the health promotion manuals, educators were able to identify and assess children with medical conditions and personal problems, and referred them to the principal. The manuals assisted the educators to guide the children to grow their own vegetables at home. The educators used the manuals to help them include children with medical conditions and physical disabilities into their physical education classes, whilst maintaining the children's health and safety. The manuals guided them on how to motivate the children to participate actively in physical activities, and the children started walking to and from school instead of using less active forms of transport. Educators had to use their own knowledge before the manual was introduced to map out the learners’ needs. One participant expressed this as follows:

‘… We sat down and studied the background of these kids … I then went to the principal and made him aware of what is happening in the classroom regarding some of our children.’ (Participant 1)

Before the introduction of the manual, the educators had to study the background of the children before they could help them:

‘But now after the manual, I realised that instead of giving them fish, it is better to teach them how to catch the fish.’ (Participant 6)

Before the arrival of the manual, learners with medical conditions, physical disabilities, and pregnancy were not included in any of the physical activities at the school. One participant expressed this situation as follows:

‘Before we received this material, we were taking the learner out of the group, he were to sit on the chair not doing anything due to their disabilities.’ (Participant 4)

However, the participants stated that after the manual was implemented these learners were no longer discriminated against during physical activities. They explained:

‘… Even the children who are disabled, they try to participate with the mild movements to their abilities. Even the hemiplegic, with the hand paralysed they try to participate. But after this manual there is life, it enlightened us to make the activity that goes in line with theme of the week …’ (Participant 4)

‘… but we are certain about their illness now and we include them … Even the child who is limping, participate with other kids as well … So whatever we do in class, the pregnant learner must be included. With us we requested a chart at the clinic that teaches us on exercises that pregnant women do. They participate in all activities.’ (Participant 3)

Although the educators aimed not to discriminate against the pregnant and disabled children, prior to the manuals, a lack of information promoted alienation of children during physical activity lessons. Hence, the manuals appeared to help bring about a positive change as the educators were prompted to include pregnant learners and learners with disabilities. In addition, with the help of the manuals, educators managed to motivate children to actively participate in physical activities and children started walking to and from school instead of using less active forms of transport. Participants expressed themselves as follows:

‘I motivated my students to walk a distance from home to school. I made them aware that it is part of physical activity. I told them that they might think that their parents do not love them by not have [*sic*] transport to school, only to find that transport maybe contributing to their unhealthiest [*sic*]. But if you walk from home to school it is part of physical activity … But after receiving this manual, it is so interesting because I now understand why we do it; even the children enjoy it as they participate for fun now … When preparing for the activity, I try to make it fun by matching them with the theme of that day or week.’ (Participant 7)

Prior to the implementation of the guidelines in the manuals, children with medical conditions and physical disabilities were not encouraged to participate in physical activities. After implementation the health promotion manuals, the educators found that they were able to involve the children and the children showed an interest and had a better understanding of health promotion activities such as physical activity.

### Meeting the nutritional needs through learner participation

Most children of Makapanstad experience poverty and a lack of food security. However, the manual provides suggestions on how this can be overcome. Prior to the manual, there was no means of assisting the children in need. Furthermore, the children associated the unemployment of their parents with poverty. An educator expressed the problem of poverty as follows:

‘They did not know that there are other means of avoiding poverty even when you are unemployed. They can also identify people who are unemployed and try to assist them not fall prey of poverty.’ (Participant 5)

Based on information included in the manuals, on food security and a need for a strategy to combat poverty, the educators started educating the children's parents about vegetable gardens. They also asked for donations from the community and gave what they collected to needy children. Furthermore, they worked with the school health promoter to establish vegetable gardens at the schools. This was described by the educators as follows:

‘I started to tell them about the food gardening, and some of them started realising the importance of food gardening. This was after I’ve [*sic*] read the manual. I started realising the importance of vegetable and fruits, the importance of it … I started to realise the importance of certain nutrients.’ (Participant 6)

‘I started to tell them about the food gardening, and some of them started realising the importance of food gardening … the B-germ [*a non-governmental* (NGO)] group, who are helping us to decrease level [*sic*] of poverty in our schools, like asking for donation [*sic*] and give to those who are really in need of them.’ (Participant 8)

Hence, the health promotion programme manuals were used by the participants to empower both the learners and their parents on how they can work towards ensuring their own food security.

### Promoting health in schools by empowering educators to teach sex education

Before the introduction of the health promotion manuals, educators lacked skills on health promotion topics such as HIV, AIDS, abuse and abstinence. The educators also had a dilemma regarding when to introduce topics such as teenage pregnancy, family planning and sex education to primary school children:

‘Before we received these documents, we had no idea in how to deal with topics like HIV and AIDS which of course we are dealing with it in primary [*school*]. Especially in life orientation, we deal a lot with topic [*sic*] like HIV and AIDS, abuse, abstinence and others.’ (Participant 2)

The participants attributed the high incidence of teenage pregnancy to poverty stating that:

‘Now the topics such as teenage pregnancy and family planning, we thought that in primary [*school*] they are not meant for the primary children … if [*we*] start telling them about family planning I will be encouraging them to become sexually active.’ (Participant 4)

But after the health promotion manuals were introduced they could see the impact of health promotion at their schools. Educators felt that they now knew the right time to introduce sex education and how to deal with pregnant children:

‘Yes we do see the impact as with teenage pregnancy, children have been guided on family planning and we see it reducing … but ultimately we realised that all these topics are suitable for all age groups irrespective of the standard of school.’ (Participant 3)

The educators stated that they had started creating awareness of health promotion topics that were in line with the DoH's provisions and invited speakers to address the school during the campaigns:

‘Every year we celebrate the HIV Awareness Day and we talk about nutrition and hygiene … I also think it helps a lot in health promotion, because I have seen that other schools do envy us especially when we celebrate the awareness days.’ (Participant 8)

However, there was also collaboration with on-going community structures. Collaboration with the civil society, such as the Love Life peer educators, was established to help curtail teenage pregnancy rates. The community health worker who was appointed by the university facilitated the programme involving the Love Life peer educators. Though the rate of teenage pregnancy has remained high, the peer educators took an initiative to start educating children on the effects of teenage pregnancy and how to prevent it:

‘With the help of the B-germ [*NGO*] group, there are some days especially the Wednesday, they address the children about teenage pregnancy. When they are addressing the children, they emphasise the good things about abstinence.’ (Participant 7)

During sex education sessions teachers were empowered to use peer education on abstinence in the prevention of teenage pregnancy.

### Social problems and abuse

Before the arrival of the health promotion manuals, the majority of educators found it very difficult to identify children who had multiple social problems. One reason was that the educators expected social workers to deal with social problems. However, after the introduction of the health promotion manuals, the educators understood their role better regarding social problems and were able to identify children experiencing different types of abuse. These included physical, verbal, sexual, and drug or alcohol abuse that had a negative impact on their learning abilities. Hence, the educators were able to refer the children who were being abused to the Department of Social Development (DSD). The educators expressed a lack of community participation regarding abuse and how it was managed. Participants stated that:

‘Another thing that we want added to the manual is the issue of abuse. There is a lot of abuse that is happening in our community. Children are abused by their uncles, by their parents in many ways. Most of them are given something as a reward, they think that it's out of love. If we start teaching them about abuse at an early stage, they can really be aware of abuse in an early stage …’ (Participant 5)

Hence, the participants emphasised that the content on abuse is lacking and should be added to the health promotion programme manual to assist the educators to help prevent abuse in the community.

## Discussion

The results of this study consisted of two themes which were underpinned by five sub-themes. These were:

1. lack of health promotion programme manuals
2. the roles of educators in health promotion
3. meeting the nutritional needs through learner participation
4. promoting health in schools by empowering educators to teach sex education
5. added roles and responsibilities of teachers.

### Lack of health promotion manuals

It was evident that before the introduction of health promotion programme manuals educators worked with few resources to address some of the adolescents’ reproductive health issues. The participants mentioned that they relied primarily on common sense and they struggled to prepare lesson plans as the health promotion themes were not part of the LO subject. Learners in schools showed limited interest in health promotion activities. This fact was evidenced by the persistent high rates of teenage pregnancy, poverty, environmental neglect and the high risk sexual behaviours. Such limited interest in health promotion activities was confirmed in a study by Peu *et al.* (2010) regarding the health education training needs of educators. The educators in this study mentioned that they lacked basic knowledge and resources regarding the presentation of health education to the learners. Indeed, Peu *et al.* (2010) state that health education packages should be developed and validated to assist educators to empower learners. Following the introduction of the manuals, educators mentioned that these manuals were a crucial resource in providing health education about healthy lifestyles. It was further mentioned that the manuals gave them confidence to present topics such as sexual health in primary school. After the introduction of the manual, the educators also observed positive changes in learner behaviour, for example, more children walked to school and took an active interest in promoting the school vegetable garden.

It came up very strongly from the results of this study that before the development of the health promotion manuals, preparation of lessons was difficult. However, immediately after implementation of manuals, the preparation of lessons became easy. This was confirmed by Panday *et al.* (2009) who attested that the introduction of health promotion guideline assisted educators with the prevention and management of learner teenage pregnancy. The aim of the guideline was to introduce peer education programmes as a strategy to combat HIV and AIDS amongst young people. In addition, peer educators would serve as role models for healthy behaviour, identifying youth who needed assistance and making necessary referrals and serving as advocates to secure resources for themselves and their peers (Panday *et al.* 2009). In the guideline it was recommended that sex education should be included in LO, HIV and AIDS programmes and peer education as strategies to reduce teenage pregnancies in South African schools (Panday *et al.* 2009). One of the WHO's objectives of health promotion programmes is to improve health care seeking behaviour in communities to enhance prompt referral to relevant professionals (WHO 2009). Most high risk behaviours, such as smoking or physical inactivity, that affect people in adulthood begin in adolescence and, therefore, adolescent interventions can help prevent such health problems (WHO 2009).

### The roles of educators in health promotion

The educators had limited knowledge on the effects of poverty on children and on how to deal with children coming from poor families. After utilising the health promotion manuals, educators were empowered to assess children with medical conditions and personal problems and referred them to the principal. In a study by Mulaudzi and Peu (2014:5) on communal child rearing, it was emphasised that the role of educators is to promote and sustain health, improve the physical and social environment where children learn and develop, and improve the children's capacity to become and stay healthy. Educators mentioned that the manuals helped them to assist children to grow their own vegetables at home. The manuals also guided them on how to include children with medical conditions and physical disabilities in physical education classes whilst maintaining their health and safety.

Educators play an important role in the health promotion of learners by preparing and presenting lessons on disease prevention and health promotion. Educators are expected to teach sex education, including prevention of teenage pregnancy, HIV and AIDS and the educators deemed this a challenge. In order for educators to guide learners on how to prevent pregnancy and contracting sexually transmitted infections, a positive and respectful approach is required to sexual health (Inman *et al.* 2011). Programmes that have had an impact on improving behaviour of children are those that were curriculum-based and led by either educators or other adults, such as health workers (Panday *et al.* 2009). The manual assisted the educators to use this approach.

### Meeting the nutritional needs through learner participation

Meeting the nutritional needs of children is one of the objectives of the school health promotion programme. The food supply needs to be sustained and promoting vegetable and fruit gardening becomes the most affordable means to ensure food security. In the Integrated SHP (National Department of Health and National Department of Basic Education 2012), it is emphasised in the school health package that nutritional assessments should be conducted on the learners. The school health promotion programme needs to address the needs of all age groups. Therefore, educators need to be made aware of the needs of school children. Celebration of awareness days is a means of community involvement and fosters community participation.

### Promoting health in schools by empowering educators to teach sex education

The participants revealed that before the introduction of the health promotion manuals, educators lacked a variety of knowledge and skills on how to prepare health education about HIV, AIDS, emotional abuse, physical abuse, sexual abuse and abstinence. The educators had a dilemma about when to introduce topics such as teenage pregnancy, family planning and sex education to primary school children. This dilemma is in line with the results of the study by Peu *et al.* (2010:36) on the health education training needs of educators. The results of this study reflected the following needs as priorities for health promotion:

- teenage pregnancy
- family planning
- HIV and AIDS
- STDs (Lavin & Cox 2012:463; Peu *et al.* 2010).

Lavin and Cox (2012:463) state that although pregnancy is a normal development process amongst women, on the other hand it has several impacts on the physical, social and psychological development of adolescents. In this study, after the introduction of the health promotion manuals, the educators could see the impact of health promotion at their schools. They felt that they now knew the right time to introduce sex education and how to deal with pregnant children. These educators stated that they had started creating awareness of health promotion topics that were in line with the Integrated SHP (DoH & DBE 2012) and invited speakers to address the school during the campaigns. Lavin and Cox (2012:467) confirmed that multicomponent programmes are necessary to address motivation and skills building with reference to comprehensive sex education programmes. These programmes provided interventions leading to a decline in teenage pregnancy of 50%.

### Social problems and abuse

Emotional, physical and sexual abuse have a serious impact on the educational outcomes of learners. Successful collaboration between the educators, parents, DSD and DoH could yield positive results in combating the causes and effects of abuse. A learner's emotional health is critical in promoting academic and lifetime success, and if left untreated, mental health disorders may lead to suicide, violence, dropping out of school, and alcohol and drug abuse (Centers for Disease Control and Prevention [CDC] 2012; Inman *et al.* 2011). Parents serve as the primary socialising agent and trusted source of information of children (Panday *et al.* 2009). In the CDC (2012:7) it was noted that ‘parent engagement in school can promote positive health behaviors among children and adolescents’. It was further mentioned that ‘students who are supported by their parents are less likely to experience emotional distress, practice unhealthy eating behaviors or attempt suicide and disengage from school and learning’. Therefore, to promote positive health behaviours amongst school children, educators need to demonstrate to parents how their children's health and education can be enhanced by their engagement in school health activities (CDC 2012).

### Limitations of the study

The results of this study are only limited to the four schools of the Makapanstad Health Promoting Schools. As a result, the findings of this study cannot be generalised to the broader population. Future studies should include a bigger sample and cover a wider area in the North- West Province. The study was limited to the views of educators. The views of the community and the learners were not taken into consideration. Having more than one focus group that included community members would have yielded better results.

### Recommendations

The following recommendations emerged from the study:

- The educators requested the university to facilitate the involvement of social workers to help with social challenges faced by children.
- The educators need support to deal with challenges such as the children's behavioural and emotional problems.
- The educators requested that a nutrition programme should be extended to the community, to correct the nutritional challenges from home.
- The educators requested a psychotherapeutic intervention, on children affected by any form of abuse, and educating other children about how to avoid becoming victims of abuse.
- Disseminating this information to other South African schools will be beneficial to the communities and the country by empowering educators to promote health in schools.

## Conclusion

In order to promote positive health behaviours amongst school children, educators need to ‘demonstrate to parents how their children's health and education can be enhanced by their engagement in school health activities’ (CDC 2012:7). The ‘school efforts to promote health among students have been shown to be more successful when parents are involved’ (7). Successful collaboration between the educators, parents, the DSD and DoH could yield positive results in combating the causes and effects brought about by unhealthy reproductive health issues. The referral and reporting of social problems, such as abuse, was undertaken by these parties as collaboration was emphasised and their roles were clearer. Introducing health promotion materials to the schools served as a point of departure for developing educational skills amongst the educators and creating a supportive environment for health in schools. Learners started home gardening to provide a food source for their families. The health promotion manual, therefore, assisted the educators to access health promotion information and to transfer this knowledge to learners, irrespective of their health status.
